# Investigation on Legal Problems Encountered by Emergency Medicine Physicians in Turkey

**DOI:** 10.1371/journal.pone.0127206

**Published:** 2015-05-18

**Authors:** Afsin Emre Kayipmaz, Cemil Kavalci, Betul Gulalp, Ummu Gulsum Kocalar, Tufan Akin Giray, Hasan Yesilagac, Betul Akbuga Ozel, Elif Celikel, Ozlem Karagun

**Affiliations:** Department of Emergency, Baskent University Faculty of Medicine, Ankara, Turkey; Central South University, CHINA

## Abstract

**Background:**

Medicine is a profession that carries certain risks. One risky area of practice is the emergency department. Emergency physicians diagnose and treat a high volume of patients, and are also responsible for preparing reports for forensic cases. In this study, we aim to investigate emergency physicians’ legal-administrative problems and reveal their level of understanding on forensic cases.

**Methods:**

An electronic questionnaire form was prepared after the approval of an ethical committee. This form was sent to the residents, specialists and academicians of emergency medicine by e-mail. The physicians were asked to fill out the form online. All the gathered data was analyzed. Descriptive statistics were presented as frequency percentages with mean and standard deviation. Chi-square tests were used to compare the groups. Correlation between number of complaint cases and age, sex, career, institution, and duration of service in emergency department were investigated. p<0.05 was considered statistically significant.

**Results:**

294 physicians participated in the questionnaire. According to the questionnaire, 170 of the physicians were reported to the patient communication units due to medical malpractice. Mean number of compliant reports was 3.20±3.5. 29 of the physicians received administrative penalties. 42 of the physicians were judged in the court for medical malpractice. 1 physician was fined 5000 Turkish Liras as a result of these judgments.

**Conclusion:**

We found that the number of complaint reports is negatively correlated with duration of service in emergency medicine and age. There was a significant difference between number of complaint reports and career (p<0.05). The physicians’ level of awareness on forensic cases was found to be insufficient. Lack of legislation knowledge may be an important cause of complaint reports concerning emergency physicians, who have a high load of patients. Thus, we think that increasing the frequency of post-graduate education sessions and periodical reviews might be beneficial.

## Introduction

According to “The Universal Declaration of Human Rights,” every single individual has the right to medical care [1]. The concept of “Patient Rights” originates from this document. Patient rights were mentioned for the first time in United States of America in the 1970s [2]. The Declaration of Lisbon, which was adopted in 1981 by World Medical Association, is the first document to outline patient rights [3]. In Turkey, “Regulations on Patient Rights” was first published in Official Gazette of the Republic of Turkey in 1998. In 2014 an updated final form was released. The regulations define the rights of patients and the rules patients must obey. Additionally, the document includes regulations on patient communication and development of patient rights councils [4, 5]. For patient communications, the Republic of Turkey Ministry of Health founded a Communication Center (SABIM) in 2004. Patient complaints can be reported to SABIM by phone via the “Alo 184” line, online, or in person at a patient communication unit. Bostan et al. reported that 36640 complaints were reported to the “Alo 184” line between years 2004 and 2009 [6].

In addition to the Regulations on Patient Rights, the Turkish Penal Code (TPC) created other regulations in 2005. The 83^rd^ law in TPC states that an “individual who kills by act of omission might be sentenced to a penalty of imprisonment for a term of 10 to 25 years”. The 22^nd^ law in TPC defines the penalties for crimes by unintentional and intentional negligence [7]. Yilmaz et al. states that since this law was adopted, there has been a significant increase in defensive medical practices of physicians in surgical specialties [8].

Medicine is a profession that carries certain risks. One risky area of practice is the emergency department (ED), which operates 24 hours a day. Patients with minor or less urgent symptoms cause unnecessary patient load when they present to the ED [9]. In order to prioritize the patients in the ED, various systems of triage are used [10]. Because of these systems, patients with less urgent complaints have longer waiting time, causing patient dissatisfaction [11].

Emergency Medicine physicians diagnose and treat a high volume of patients, and are also responsible for preparing reports for forensic cases. Unexpectedly, Gunaydın et al. reported that 70.4% of emergency medicine physicians, who have a mean 8.5 years of service, do not fully understand the physician responsibility for preparing forensic reports [12].

In this study, we aim to investigate emergency medicine physicians’ legal-administrative problems and reveal their level of understanding on forensic cases.

## Materials and Methods

This study was approved by the Medical and Health Sciences Research Ethics Committee of Baskent University (Project Number: KA14/352). An electronic questionnaire form was prepared (Table 1). This form was sent to the residents, specialists and academicians of emergency medicine by e-mail. The physicians were asked to fill the form online. Participation was voluntary. Access to the electronic form was limited to 10 days. Data was collected anonymously. At the end of the 10-day period, all the gathered data was analyzed in SPSS for Windows, version 13. Descriptive statistics were presented as frequency percentages with mean and standard deviation. Chi-square tests were used to compare the groups. Correlation between number of complaint cases and age, sex, career, institution, and duration of service in emergency department were investigated. p<0.05 was considered statistically significant.

**Table 1:** Questions and Answers of Electronic Questionnaire.

Questionnaire
**1) Age**
**2) Sex** (Female/Male)
**3) Career** (Resident/ Emergency Medicine Specialist/ Academician)
**4) Institution** (Private Hospital/State Hospital/Training and Research Hospital of Ministry of Health (TRH)/Hospital of Private University/Hospital of State University/Other)
**5) Years of service in Emergency Medicine** (1-4/5-9/10-14/15 and above)
**6) Have you ever been reported to patient communication units because of medical malpractice?** (Yes/No)
**7) If your answer to the previous question is “Yes”, how many times you have been reported?**
**8) Have you received any administrative penalty because of these reports?** (Yes/No)
**9) If your answer to the previous question is “Yes”, what was the type of the penalty?** (Verbal Warning/Written Warning/Condemnation/Salary Cut/Halt of Career Progression)
**10) How many times did you receive these penalties?** (Number of Verbal Warnings/Number of Written Warnings/Number of Condemnations/Number of Salary Cuts/Number of Halt of Career Progression)
**11) Have you ever been judged in the court because of medical malpractice?** (Yes/No)
**12) If your answer to the previous question is “Yes”, have you ever received any legal penalties?** (Yes/No)
**13) If your answer to the previous question is “Yes”, what was the type of the penalty?** (Criminal Fines/Imprisonment)
**14) If you have received criminal fines or imprisonment, what was the amount or duration?** (Amount of Criminal Fines/Duration of Imprisonment)
**15) Which of the following cases are forensic cases?** (Burns, industrial accidents, violence against women, admissions dead on arrival to the hospital, child abuse, claim and possibility of human rights violations, bee sting, scorpion sting, elder abuse, elder neglect, abandoned individuals, insulin overdose of patient with diabetes, fall from 4^th^ floor as a result of slipping, explosion of pressure cooker, splatter of blood of the hepatitis c positive patient to the eye of healthcare professional) **(Correct answer: All of them)**
**16) A conscious patient who attempted to suicide by taking 40 tablets of Sertraline 25 mg refuses treatment and wants to leave the emergency department. What should the physician do?** (1. Patient must be discharged after signing form for refusal of treatment. / 2.Prosecutorship must be informed and their help on forced treatment must be sought. / 3. Patient must be handed over to his/her friends after they sign the form for refusal of treatment. / 4. Mother, father, sibling, spouse or child of the patient must be called, if they approve the refusal by signing it, the patient must be handed over to them. / 5. Physician should wait until the patient loses consciousness, then the physician must start the treatment.) **(Correct answer: 4/5)**
**17) An adult, mentally sane patient admitted to the emergency department refuses the treatment but his/her relatives accepts treatment. What should the physician do?** (Patient must be treated by force/Patient must be informed on possible risks. His written consent, stating that he/she understood the risks, must be obtained and the patient must be discharged.) **(Correct answer: Patient must be informed on possible risks. His written consent stating that he/she understood the risks must be obtained and the patient must be discharged.)**
**18) Which of the following are not eligible for the consent of the patient?** (Patient, mother, father, sibling, spouse, brother-in-law, cousin, aunt, colleague, neighbor, lover) **(Correct answer: Brother-in-law, cousin, aunt, colleague, neighbor, lover)**
**19) Have legal/administrative investigation progresses influenced your medical decisions?** (Yes/No)
**20) Do you think you have received enough support from your institution when you encountered legal investigations?** (Yes/No)

## Results

294 physicians participated in the questionnaire. 208 physicians were female and 86 were male. The mean age of participants was 34.33±5.55. Titles, institutions and duration of service in emergency medicine are summarized in Table 2.

**Table 2:** Career, institution and duration of service in emergency medicine.

Categorical		Number (n)
Career	Resident	126
	Emergency Medicine Specialist	97
	Academician	71
Institution	Private Hospital	6
	State Hospital	53
	Training and Research Hospital (TRH)	109
	Hospital of Private University	33
	Hospital of State University	86
	Other	7
Duration of Service in Emergency Medicine	1–4	95
	5–9	116
	10–14	52
	15 and above	31

According to the questionnaire, 170 of the physicians were reported to the patient communication units due to medical malpractice. Mean number of compliant reports was 3.20±3.5. 29 of the physicians received administrative penalties. 2 received both verbal and written warning and 1 received both verbal warning and condemnation. Number of physicians receiving administrative penalties and number of penalties by career are summarized in Table 3.

**Table 3:** Number of physicians receiving administrative penalties and number of penalties by career.

Categorical			Number (n)
Type of administrative penalty	Verbal warnings	14 physicians	1
		3 physicians	2
		1 physician	3
		1 physician	15
	Written warnings	8 physicians	1
	Condemnation	3 physicians	1
	Salary deduction	1 physician	1
	Halt of career progression	1 physician	1
Reported faculty staff		**Number of compliant reports/Total number of physicians (n)**	
	Resident	61/126	
	Emergency medicine specialist	67/97	
	Academician	42/71	

42 of the physicians were judged in the court for medical malpractice. According to the questionnaire, 1 physician was fined 5000 Turkish Liras as a result of these judgments.

24 physicians (8.2%) correctly answered the question “Which of the following cases are forensic cases?” 47 physicians (16%) correctly answered the question about the patient who attempted to suicide by taking Sertraline and refused the treatment. 104 physicians (35.4%) correctly answered the question about the adult, sane patient refusing treatment. Only 10 physicians (3.4%) correctly answered the question about eligible persons for consent.

The number of compliant reports was negatively correlated with years of service in emergency department and age (r = -0.116, p = 0.048 and r = -0.131, p = 0.025, respectively). Number of complaints statistically differed with career (p = 0.008). 122 of the physicians (41.5%) think that legal or administrative investigation progresses influenced his/her medical decisions, and 59 physicians (20.1%) think they received enough support from their institutions.

## Discussion

We found that the number of complaints is negatively correlated with age and duration of service in emergency. There was a significant difference between number of complaint reports and career (p = 0.008). The physicians’ level of awareness on forensic cases was found to be insufficient.

Medicine is a profession that carries risks. ED is one risky area of practice. Moreover, emergency physicians have to examine a high volume of patients, and are also responsible for preparing reports for forensic cases. We demonstrate emergency physicians’ legal-administrative problems and their level of understanding on forensic cases is insufficient. This results support efficient training and experience help avoiding legitimate problems.

According to the questionnaire, the negative correlation between number of compliant reports with years in service and age revealed that experience is an important factor for working in ED. We determined that as participant’s age and duration of service in ED increases, the number of compliant reports decreases. Experience rises through age and practice. We believe that physicians’ previous negative experiences force them to be more careful and mindful. However, young and inexperienced physicians may act in an excited and impatient way because of the stressful atmosphere of ED. Thus, necessary attention to the patients may not be paid. Besides, lack of knowledge and misestimated consequences are the increasing factors of complaints as well. Our evidences support that the efficient approach to the patient and effective communication with them are the skills that are acquired in the course of time. Akkaya et al. similarly reported that medical experience of emergency medicine physicians impacts patient satisfaction [13].

In Turkey, “Forensic Medicine Internship” takes part in the undergraduate core curriculum. The topics covered in this internship include risk management, malpractice, legal responsibilities, poisonings, suicide ideation/attempts, neglect/abuse and violence [14]. The lecturers of forensic medicine department instruct about these subjects. At the same time, core curriculum of emergency medicine residency contains lectures about medical evidences, medico-legal situations and related legislation/regulation [15]. These topics are also mentioned in clinical trainings and in-service seminars as well. According to the survey results, we believe that it would be beneficial for these educations to be repeated frequently. Besides, it would be wise to organize special sessions of legal problems in symposiums and congresses.

Only 8.2% of the participants correctly answered question 15 (about forensic cases). Similarly, 16% correctly answered question 16 (the suicide case). According to “The Universal Declaration of Human Rights”, bodily integrity is a fundamental right [1]. Without the approval of the patient, no treatment can be undertaken. Even though the case of the patient in question 16 may be considered as committing a suicide, unless he is unconscious, he has the right to refuse any treatment. If the patient is deficient in identifying reality, he should not be discharged from the emergency service on his/her own. First degree relatives should be invited to the hospital to consider the patient’s condition. 35.4% correctly answered question 17 (patient refusing treatment). According to this question, if the conscious adult patient refuses the treatment even though his family approves it, the request of patient should be applied. 3.4% correctly answered question 18 (eligible persons for consent). The unauthorized peoples’ consent is legally invalid. The physicians’ lack of knowledge about this topic may lead to malpractice lawsuits. As it is suggested above, this problem may be solved by frequent seminars.

According to these results, the physicians who participated in this questionnaire have a low level of awareness on forensic cases. From this result, we conclude that one of the factors resulting in complaint reports is ignorance of legislation. In a study investigating physician level of awareness on patient rights in a university hospital, Yurumez et al. found a sufficiently high level of awareness [16]. However, they emphasized that the issue of patient rights must be kept on the agenda. Increasing the frequency of post-graduate education sessions and periodical review courses may aid in this cause. Yilmaz et al. also reported that educating healthcare professionals on patient rights is beneficial [17].

There was a significant difference between the number of compliant reports and career according to the questionnaire. While 48% of residents were reported, the rate increased to 59% for faculty staff and 69% for attending physicians. Faculty staffs share responsibility for residents, explaining the high report rate. Attending physicians of emergency medicine have the highest rate of reports. Tekwani et al. reported that patient load of the emergency department significantly reduces patient satisfaction [18]. This could be explained with the fact that attending physicians of emergency medicine encounter patients alone, especially at institutions with high patient load.

## Conclusion

As a result of our study, we found that the number of complaint reports is negatively correlated with duration of service in emergency medicine and age. There was a significant difference between number of complaint reports and career. The physicians’ level of awareness on forensic cases was found to be insufficient.

Lack of legislation knowledge may be an important cause of complaint reports concerning emergency department physicians, who have a high load of patients. Thus, we think that increasing the frequency of post-graduate education sessions and periodical reviews might be beneficial.
